# Supplementary material for: Structural basis of thiamine transport and drug recognition by SLC19A3
Source: Nat Commun. 2024 Oct 2;15:8542. doi: 10.1038/s41467-024-52872-8 (PMC11447181; doi:10.1038/s41467-024-52872-8)
Supplement: Supplementary file 3 — Description of Additional Supplementary Files [file 41467_2024_52872_MOESM3_ESM.docx]

**Description of Additional Supplementary Files**

**Supplementary Movie 1:** Morph between the outward-open (Nb3.4-bound, PDB 8S5U) and the inward-open (Nb3.7-bound, PDB 8S61) conformations of hSLC19A3, seen from the membrane plane. The NTD (purple) and the CTD (cyan) move as rigid domains in a rocker-switch type mechanism, creating a moving barrier around the bound substrate thiamine (orange).

**Supplementary Movie 2:** Extracellular view of Movie 1.

**Supplementary Movie 3:** Cytoplasmic view of Movie 1.

**Supplementary Data 1 File**

**Construct sequences Tab:** Names, short descriptions, and amino acid sequences of all constructs produced and used in this study.

**Primer sequences Tab:** Names, short descriptions, and nucleotide sequences of all DNA oligonucleotides (primers) used in this study.

**Mass spectrometry raw data Tab:** Sample overview and LC-MS/MS chromatograms of the cell-based thiamine-d3 uptake assays. Experiments were performed and interpreted as described in the methods section.
