# Supplementary material for: Structural basis of thiamine transport and drug recognition by SLC19A3
Source: Nat Commun. 2024 Oct 2;15:8542. doi: 10.1038/s41467-024-52872-8 (PMC11447181; doi:10.1038/s41467-024-52872-8)
Supplement: Supplementary file 8 — Reporting Summary [file 41467_2024_52872_MOESM8_ESM.pdf]

nature portfolio

Corresponding author(s): Christian Loew

Last updated by author(s): 5 September 2024

Reporting Summary

Nature Portfolio wishes to improve the reproducibility of the work that we publish. This form provides structure for consistency and transparency in reporting. For further information on Nature Portfolio policies, see our [Editorial Policies](#) and the [Editorial Policy Checklist](#).

Statistics

For all statistical analyses, confirm that the following items are present in the figure legend, table legend, main text, or Methods section.

n/a	Confirmed	
[ ]	[x]	The exact sample size (*n*) for each experimental group/condition, given as a discrete number and unit of measurement
[ ]	[x]	A statement on whether measurements were taken from distinct samples or whether the same sample was measured repeatedly
[x]	[ ]	The statistical test(s) used AND whether they are one- or two-sided<br>*Only common tests should be described solely by name; describe more complex techniques in the Methods section.*
[x]	[ ]	A description of all covariates tested
[x]	[ ]	A description of any assumptions or corrections, such as tests of normality and adjustment for multiple comparisons
[x]	[ ]	A full description of the statistical parameters including central tendency (e.g. means) or other basic estimates (e.g. regression coefficient) AND variation (e.g. standard deviation) or associated estimates of uncertainty (e.g. confidence intervals)
[ ]	[x]	For null hypothesis testing, the test statistic (e.g. *F*, *t*, *r*) with confidence intervals, effect sizes, degrees of freedom and *P* value noted<br>*Give P values as exact values whenever suitable.*
[x]	[ ]	For Bayesian analysis, information on the choice of priors and Markov chain Monte Carlo settings
[x]	[ ]	For hierarchical and complex designs, identification of the appropriate level for tests and full reporting of outcomes
[x]	[ ]	Estimates of effect sizes (e.g. Cohen's *d*, Pearson's *r*), indicating how they were calculated

Our web collection on [statistics for biologists](#) contains articles on many of the points above.

Software and code

Policy information about [availability of computer code](#)

Data collection	The Cryo-EM data were collected using EPU (Thermo Fisher Scientific);<br>The Thermal Stability data were collected using PR. ThermControl (version 2.1.2);<br>Biolayer interferometry data were collected using the Octet System Data Acquisition v11.0 (FortéBio).<br>Mass spec data were analyzed with TraceFinder 5.1.
Data analysis	The Cryo-EM data were processed with CryoSPARCv4.4 and 4.5;<br>Model building was performed with Coot v. 0.9.8.1;<br>Thermal Stability data were analyzed using MoltenProt;<br>Figures were prepared using UCSF ChimeraX (v. 1.6.1);<br>Biolayer interferometry data were analyzed using the Octet Data Analysis software v10.0 (FortéBio);<br>MapQ has been used for MS data, available on GitHub: https://github.com/gregdp/mapq.

For manuscripts utilizing custom algorithms or software that are central to the research but not yet described in published literature, software must be made available to editors and reviewers. We strongly encourage code deposition in a community repository (e.g. GitHub). See the Nature Portfolio [guidelines for submitting code & software](#) for further information.

Data

Policy information about availability of data

All manuscripts must include a data availability statement. This statement should provide the following information, where applicable:

- Accession codes, unique identifiers, or web links for publicly available datasets
- A description of any restrictions on data availability
- For clinical datasets or third party data, please ensure that the statement adheres to our policy

Structural models and cryo-EM density maps have been deposited in the PDB and the EMDB under the following accession codes: hSLC19A3-wt:Nb3.4-apo (8S4U.pdb, EMD-19716); hSLC19A3-wt:Nb3.4:thiamine (8S5U.pdb, EMD-19750); hSLC19A3-wt:Nb3.3-apo (9G5K.pdb, EMD-51088); hSLC19A3-gf:Nb3.7:thiamine (8S61.pdb, EMD-19754); hSLC19A3-gf:Nb3.7:Fedratinib (8S5W.pdb, EMD-19752); hSLC19A3-gf:Nb3.7:Amprolium (8S62.pdb, EMD-19755), hSLC19A3-gf:Nb3.7: Hydroxychloroquine (8S5Z.pdb, EMD-19753).

All reagents generated in this study are available from the Lead Contact with a completed Materials Transfer Agreement.

Research involving human participants, their data, or biological material

Policy information about studies with human participants or human data. See also policy information about sex, gender (identity/presentation), and sexual orientation and race, ethnicity and racism.

Reporting on sex and gender	This information has not been collected because human research participants were not involved in this study
Reporting on race, ethnicity, or other socially relevant groupings	see above
Population characteristics	see above
Recruitment	see above
Ethics oversight	see above

Note that full information on the approval of the study protocol must also be provided in the manuscript.

Field-specific reporting

Please select the one below that is the best fit for your research. If you are not sure, read the appropriate sections before making your selection.

☒ Life sciences ☐ Behavioural & social sciences ☐ Ecological, evolutionary & environmental sciences

For a reference copy of the document with all sections, see nature.com/documents/nr-reporting-summary-flat.pdf

Life sciences study design

All studies must disclose on these points even when the disclosure is negative.

Sample size	Sample sizes were triplicates. No statistical methods were used to determine the sample size. They were, however, used to calculate standard deviations and p-values.
Data exclusions	No data were excluded.
Replication	Shown data typically represent the mean ± SD of three biological replicates.
Randomization	For the biochemical and structural measurements performed in this study, randomisation is not applicable.
Blinding	For the biochemical and structural measurements performed in this study, blinding is not applicable.

Reporting for specific materials, systems and methods

We require information from authors about some types of materials, experimental systems and methods used in many studies. Here, indicate whether each material, system or method listed is relevant to your study. If you are not sure if a list item applies to your research, read the appropriate section before selecting a response.

Materials & experimental systems

n/a	Involved in the study
☐	☒ Antibodies
☐	☒ Eukaryotic cell lines
☒	☐ Palaeontology and archaeology
☒	☐ Animals and other organisms
☒	☐ Clinical data
☒	☐ Dual use research of concern
☒	☐ Plants

Methods

n/a	Involved in the study
☒	☐ ChIP-seq
☒	☐ Flow cytometry
☒	☐ MRI-based neuroimaging

Antibodies

Antibodies used	Three nanobodies (VHH fragments) were used in this study. We generated them ourselves, as described in the manuscript.
Validation	The specificity and binding affinities of the nanobodies were validated using ELISAs and BLI.

Eukaryotic cell lines

Policy information about cell lines and Sex and Gender in Research

Cell line source(s)	Expi293F™ (ThermoFischer) and Expi293F™ GnTI- (ThermoFischer)
Authentication	No authentication of the cell lines was done by the authors.
Mycoplasma contamination	We confirm that all cell lines tested negative for mycoplasma contamination.
Commonly misidentified lines (See ICLAC register)	No commonly misidentified cell lines were used.

Plants

Seed stocks	N/A
Novel plant genotypes	N/A
Authentication	N/A
